# Hypoxia/Reoxygenation Effects on Ion Transport across Rat Colonic Epithelium

**DOI:** 10.3389/fphys.2016.00247

**Published:** 2016-06-21

**Authors:** Sabine Schindele, Ervice Pouokam, Martin Diener

**Affiliations:** Institute of Veterinary Physiology and Biochemistry, University GiessenGiessen, Germany

**Keywords:** Cl^−^ secretion, electrolyte transport, intracellular Ca^2+^, rat colon

## Abstract

Ischemia causes severe damage in the gastrointestinal tract. Therefore, it is interesting to study how the barrier and transport functions of intestinal epithelium change under hypoxia and subsequent reoxygenation. For this purpose we simulated hypoxia and reoxygenation on mucosa-submucosa preparations from rat distal colon in Ussing chambers and on isolated crypts. Hypoxia (N_2_ gassing for 15 min) induced a triphasic change in short-circuit current (I_sc_): a transient decrease, an increase and finally a long-lasting fall below the initial baseline. During the subsequent reoxygenation phase, I_sc_ slightly rose to values above the initial baseline. Tissue conductance (G_t_) showed a biphasic increase during both the hypoxia and the reoxygenation phases. Omission of Cl^−^ or preincubation of the tissue with transport inhibitors revealed that the observed changes in I_sc_ represented changes in Cl^−^ secretion. The radical scavenger trolox C reduced the I_sc_ response during hypoxia, but failed to prevent the rise of I_sc_ during reoxygenation. All changes in I_sc_ were Ca^2+^-dependent. Fura-2 experiments at loaded isolated colonic crypts revealed a slow increase of the cytosolic Ca^2+^ concentration during hypoxia and the reoxygenation phase, mainly caused by an influx of extracellular Ca^2+^. Surprisingly, no changes could be detected in the fluorescence of the superoxide anion-sensitive dye mitosox or the thiol-sensitive dye thiol tracker, suggesting a relative high capacity of the colonic epithelium (with its low O_2_ partial pressure even under physiological conditions) to deal with enhanced radical production during hypoxia/reoxygenation.

## Introduction

Intestinal hypoxia, e.g., caused by arterial or venous thromboembolism or mechanical compression, is a severe gastroenterologic disease associated with a high lethality (Haglund and Bergqvist, 1999). A reduction in mucosal blood flow impairs energy supply to the high energy demanding intestinal epithelium leading to severe mucosal damage and a loss of the barrier function of the epithelium. This damage may be even exaggerated after reperfusion/reoxygenation due to the production of reactive oxygen species. The generation of these oxidants begins already during the hypoxic phase due to the accumulation of hypoxanthine and the proteolytic conversion of xanthine dehydrogenase into xanthine oxidase. Hypoxanthine is oxidized to xanthine, which is then further oxidized to uric acid; both reactions lead to the production of superoxide (${\text{O}}_{2}^{-}$•). When oxygen supply is restored during reperfusion/reoxygenation, radical production is exaggerated by ${\text{O}}_{2}^{-}$• generation in mitochondria (Dröge, 2002; Gonzalez et al., 2015).

Beside radical production, a further challenge for the epithelium during the ischemic/hypoxic phase is the fall in ATP production, with severe consequences for the activity of ATP-dependent enzymes such as the Na^+^-K^+^-ATPase, which is the motor for transcellular absorption or secretion of many solutes across epithelia (Kaplan, 1985). In excitable tissues such as heart, brain or vascular smooth muscle cells, however, the fall of the ATP/ADP ratio triggers a protective mechanism, i.e., the activation of ATP-sensitive K^+^ (K_ATP_) channels. The opening of these channels hyperpolarizes the membrane and thereby decreases energy demand via reduced excitability (for review of these channels, see Hibino et al., 2010). Ionic currents sensitive to sulphonylurea derivatives (which are prototypical blockers of K_ATP_ channels), however, have also been measured across the basolateral membrane of colonic epithelium of man (Maguire et al., 1999) and rat (Hennig and Diener, 2009). These channels function as heterooctamers (Babenko et al., 1998) consisting of four pore-forming subunits (K_ir_) building an inwardly-rectifying K^+^ channel and four regulatory, sulphonylurea-binding (SUR) subunits. On the molecular level, two pore-forming isoforms (K_ir_ 6.1 and K_ir_ 6.2) and two regulatory subunits (SUR1, SUR2B) have been identified on the mRNA as well as the protein level in colonic epithelium (Pouokam et al., 2013). As these channels play a central role in the protection of excitable tissues against hypoxia (e.g., Hund and Mohler, 2011), we investigated the impact of hypoxia/reoxygenation on the barrier and ion transport functions of the intestinal epithelium and the contribution of these channels to the changes induced. Ussing chamber experiments with mucosa-submucosa preparations and imaging experiments with isolated crypts from rat colon were conducted for this investigation.

## Materials and methods

### Tissue preparation

Wistar rats of both sexes were used with a body mass of 170–220 g for imaging experiments or of 180–280 g for Ussing chamber experiments. The animals were bred and housed at the institute of veterinary physiology and biochemistry of the Justus-Liebig-University Giessen at an ambient temperature of 22.5°C and air humidity of 50–55% on a 12 h: 12 h light-dark cycle with free access to water and food until the time of the experiment. The rats were numbed by a stroke on the head and killed by cervical dislocation (approved by the named animal welfare officer of the Justus Liebig University, administrative number 487_M) and performed according to the German and European animal welfare law.

The colon was flushed with an ice-cold Tyrode solution and then put into ice-cold bathing solution. The serosa and muscularis propria were stripped away by hand to obtain the mucosa-submucosa preparation of the distal colon. Briefly, the colon was placed on a small plastic rod with a diameter of 5 mm. A circular incision was made near the anal end with a blunt scalpel and the serosa together with the tunica muscularis were gently removed in the proximal direction. Two segments of the distal colon of each rat were used for the experiments.

### Solutions

The Ussing chamber experiments were carried out in a Tyrode solution containing (in mmol·l^−1^): NaCl 140, KCl 5.4, HEPES (N-(2-hydroxyethyl)piperazine-N′-2-ethanesulfonic acid) 10, glucose 12.2, CaCl_2_ 1.25, MgCl_2_ 1. The pH was adjusted with NaOH to 7.4. For the Cl^−^-free buffer, NaCl and KCl were replaced equimolarly by Na gluconate and K gluconate. For the Ca^2+^-free buffer, CaCl_2_ was omitted. For crypt isolation, a Ca^2+^- and Mg^2+^-free Hanks's balanced solution was used containing 10 mmol·l^−1^ EDTA (ethylenediamine tetraacetic acid), pH was adjusted with tris-base (tris(hydroxymethyl)-aminomethane) to 7.4. The isolated crypts were stored in a high potassium Tyrode solution consisting of (mmol·l^−1^): K gluconate 100, KCl 30, HEPES 10, NaCl 20, MgCl_2_ 1, CaCl_2_ 1.25, glucose 12.2, sodium pyruvate 5 and 1 g·l^−1^ BSA; pH was 7.4. All solutions were either gassed with room air to mimic normoxic conditions or with N_2_ to induce hypoxia.

### Crypt isolation for imaging experiments

For the isolation of intact colonic crypts, the mucosa-submucosa preparation was fixed on a plastic holder with tissue adhesive and transferred for about 5–7 min to the EDTA solution. The tissue sample was vibrated once for about 30 s in order to isolate intact crypts. They were collected in high K^+^ Tyrode buffer, similar to the intracellular medium (Böhme et al., 1991). The isolation procedure was performed at 38°C.

The crypts were attached to the surface of a cover slip (diameter 22 mm) with the aid of poly-L-lysine (0.1 mg·ml^−1^; Biochrom, Berlin, Germany). For Ca^2+^-imaging, they were incubated for 60 min with 2.5 μmol·l^−1^ fura-2 acetoxymethylester (AM) in the presence of 0.05 g·l^−1^ pluronic acid (Life Technologies, Darmstadt, Germany). Afterwards, the dye which was not taken up by the cells was washed away. Then the preparation was transferred to a gas-tight hypoxia chamber (own design; see inset of **Figure 6**). The chamber had a volume of 2 ml. It was perfused with 140 mmol·l^−1^ NaCl Tyrode at about 5 ml·min^−1^. In order to mimic normoxic or hypoxic conditions, the superfusing solutions were continuously gassed with air or with N_2_.

Changes in the cytosolic Ca^2+^ concentration were monitored as changes in the fura-2-ratio (emission at an excitation wave length of 340 nm divided by the emission at an excitation wave length of 380 nm; emission was measured at a wave length above 410 nm). In order to measure mitochondrial production of hydrogen peroxide (H_2_O_2_), the crypts were incubated for 60 min with 5·10^−6^ mol·l^−1^ mitosox at room temperature. Potential production of superoxide anion (${\text{O}}_{2}^{-}$•) was monitored as changes in the mitosox signal (emission above 580 nm at an excitation wave length of 390 nm). For the registration of changes in the cytosolic concentration of reduced glutathione (GSH), the crypts were incubated with for 60 min with 2·10^−5^ mol·l^−1^ thiol tracker violet stain at room temperature. The GSH concentration was monitored as changes in the thiol tracker violet signal (emission above 410 nm at an excitation wave length of 380 nm).

The imaging experiments were carried out on an inverted microscope (Olympus IX-50; Olympus, Hamburg, Germany), equipped with an epifluorescence set-up and an image analysis system (Till Photonics, Martinsried, Germany). Several regions of interest (ROI's) were selected, each one with the size of one cell. Data were sampled at 0.2 Hz. The baseline in the fluorescence signal was measured for several minutes before any drug was added.

### Short-circuit current measurement

The tissue was fixed in a modified Ussing chamber, bathed with a volume of 3.5 ml on each side of the mucosa-submucosa preparation and short-circuited by a computer-controlled voltage-clamp device (Ingenieur Büro Mußler, Aachen, Germany) with correction for solution resistance. The tissue was incubated at 37°C and the electric activities were measured on an area of 1 cm^2^. Tissue conductance (G_t_) was measured every min by the voltage deviation induced by a current pulse (±50 μA, duration 200 ms) under open-circuit conditions. Short-circuit current (I_sc_) was continuously recorded on a chart recorder. I_sc_ is expressed as μEq·h^−1^·cm^−2^, i.e., the flux of monovalent ion per time and area with 1 μEq·h^−1^·cm^−2^ = 26.9 μA·cm^−2^.

### Drugs

BaCl_2_ and GdCl_3_ were dissolved in an aqueous stock solution. Bumetanide was dissolved in ethanol (final maximal concentration 0.25% v/v). 2-APB (2-aminoethoxydiphenyl borate), glibenclamide (Boehringer Mannheim, Mannheim, Germany), NPPB, pinacidil and trolox C were dissolved in dimethylsulphoxide (final maximal concentration 0.25% v/v). All fluorescent dyes were obtained from Life Technologies, (Darmstadt, Germany). If not indicated differently, drugs were from Sigma (Taufkirchen, Germany).

### Statistics

Results are given as means ± one standard error of the mean (SEM) with the number n of analyzed tissues or cells received from at least three animals. For the comparison of two groups, either a student's *t*-test or a Mann-Whitney *U*-test was used. An *F*-test decided, which test method had to be used. To compare more than two groups, an analysis of variance was performed followed by *post-hoc* Tukey's -test. *P* < 0.05 was considered to be statistically significant.

## Results

### Hypoxia and subsequent reoxygenation modulate ion transport across rat colonic epithelium

**Figure 1:**
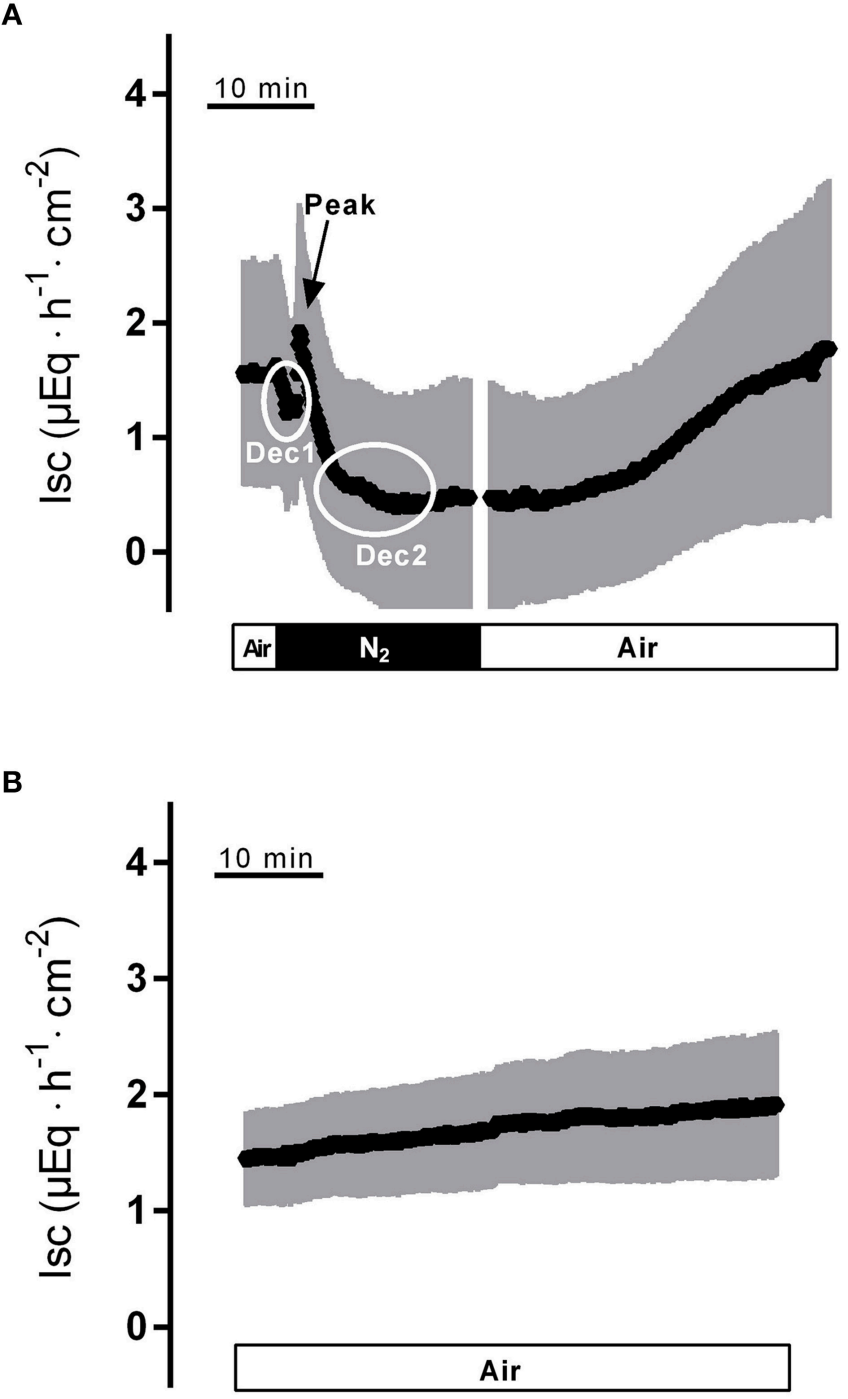
**Effect of hypoxia (N_**2**_, black bar) and reoxygenation on I_**sc**_ (A) in comparison to a time-dependent control (B) continuously gassed with air (white bar)**. Dec1, Peak and Dec2 mark the minimal respective maximal changes in I_sc_ quantified in Tables 1–3. Values are means (symbols) ± SEM (gray area), *n* = 8 **(A)** or 15 **(B)**. For statistics, see Table 1.

Hypoxia reached by means of N_2_ gassing for 15 min in Ussing chambers was preceded and followed by room air gassing in order to mimic normoxia and reoxygenation, respectively. Baseline in short-circuit current (I_sc_), which is a measure of net ion movement across the epithelium, at the end of the normoxic period amounted to 1.55 ± 0.98 μEq·h^−1^·cm^−2^ (*n* = 8). As a response to hypoxia, a triphasic change (Figure 1A) in I_sc_ was induced. It consisted of an initial, transient decrease (Dec1 in Figure 1A) of −0.91 ± 0.20 μEq·h^−1^·cm^−2^ under the initial baseline. This was followed by a transient rise (peak) of 0.57 ± 0.17 μEq·h^−1^·cm^−2^ and finally a long-lasting decrease (Dec2 in Figure 1A) of −1.26 ± 0.19 μEq·h^−1^·cm^−2^ (Table 1) under the initial baseline. Approximately 10 min after the start of the hypoxic phase, the I_sc_ reached a stable plateau (Figure 1A). Reoxygenation caused the I_sc_ to rise again after a delay of about 7 min. After 15 min in air gassing, the I_sc_ had risen by 0.37 ± 0.20 μEq·h^−1^·cm^−2^ (Table 1) compared to the baseline at the end of the N_2_ period and further rose to value of 1.69 ± 1.38 μEq·h^−1^·cm^−2^ (*n* = 8) above the current during the hypoxic period, when the reoxygenation period was extended to a duration of 30 min (Figure 1A). In time-dependent control experiments with continuous air gassing, only a slight increase in I_sc_ was observed (Figure 1B), which rose by 0.35 ± 0.2 μEq·h^−1^·cm^−2^ (*n* = 15) in the same time interval as described above for the hypoxia/reoxygenation experiments.

**Table 1:** **Effect of drugs modulating K^**+**^ channel activity on the I_**sc**_ induced by hypoxia/reoxygenation**.

**Changes in I_sc_ (ΔI_sc_) induced by hypoxia and reoxygenation (μEq·h^−1^·cm^−2^)**					
**Condition**	**Hypoxia**			**Reoxygenation**	
	**Initial decrease (Dec1) 0–5 min**	**Peak hypoxia 0–5 min**	**Secondary decrease (Dec2) 5–15 min**	**Peak reoxygenation 3–15 min**	***n***
Control	−0.91 ± 0.20	0.57 ± 0.17	−1.26 ± 0.19	0.37 ± 0.20	8
BaCl_2_	−0.54 ± 0.12	−0.15 ± 0.11^*^	−0.81 ± 0.16	0.47 ± 0.15	10
Glibenclamide	−0.80 ± 0.18	0.24 ± 0.09	−1.24 ± 0.33	0.78 ± 0.55	6
Pinacidil	−0.60 ± 0.18	0.49 ± 0.13	−1.14 ± 0.28	0.54 ± 0.14	5

*The effects of hypoxia and reoxygenation on I_sc_ were tested in presence of different K^+^ channel blockers or activators (all administered to the serosal side). Values were taken as follows (see also Figure 1): Dec1: minimal I_sc_ during min 0–5 of the hypoxic period; peak hypoxia: maximal I_sc_ during min 0–5 of the hypoxic period; Dec2: minimal I_sc_ during min 5–15 of the hypoxic period; peak reoxygenation: maximal I_sc_ during min 3–15 of the reoxygenation period. The first row shows the response to hypoxia followed by reoxygenation in the absence of inhibitors. Drugs used were: Ba^2+^ (a nonselective K^+^ channel blocker; 10^−2^ mol·l^−1^), glibenclamide (an inhibitor of K_ATP_ channels; 5·10^−4^ mol·l^−1^) and pinacidil (an opener of K_ATP_ channels; 5·10^−4^ mol·l^−1^). Data are given as change of I_sc_ (ΔI_sc_) compared to the baseline just prior the start of the respective gassing period and are means ± SEM. n = number of tissues*.

* *P < 0.05 vs. control (analysis of variances followed by post-hoc test of Tukey)*.

**Figure 2:**
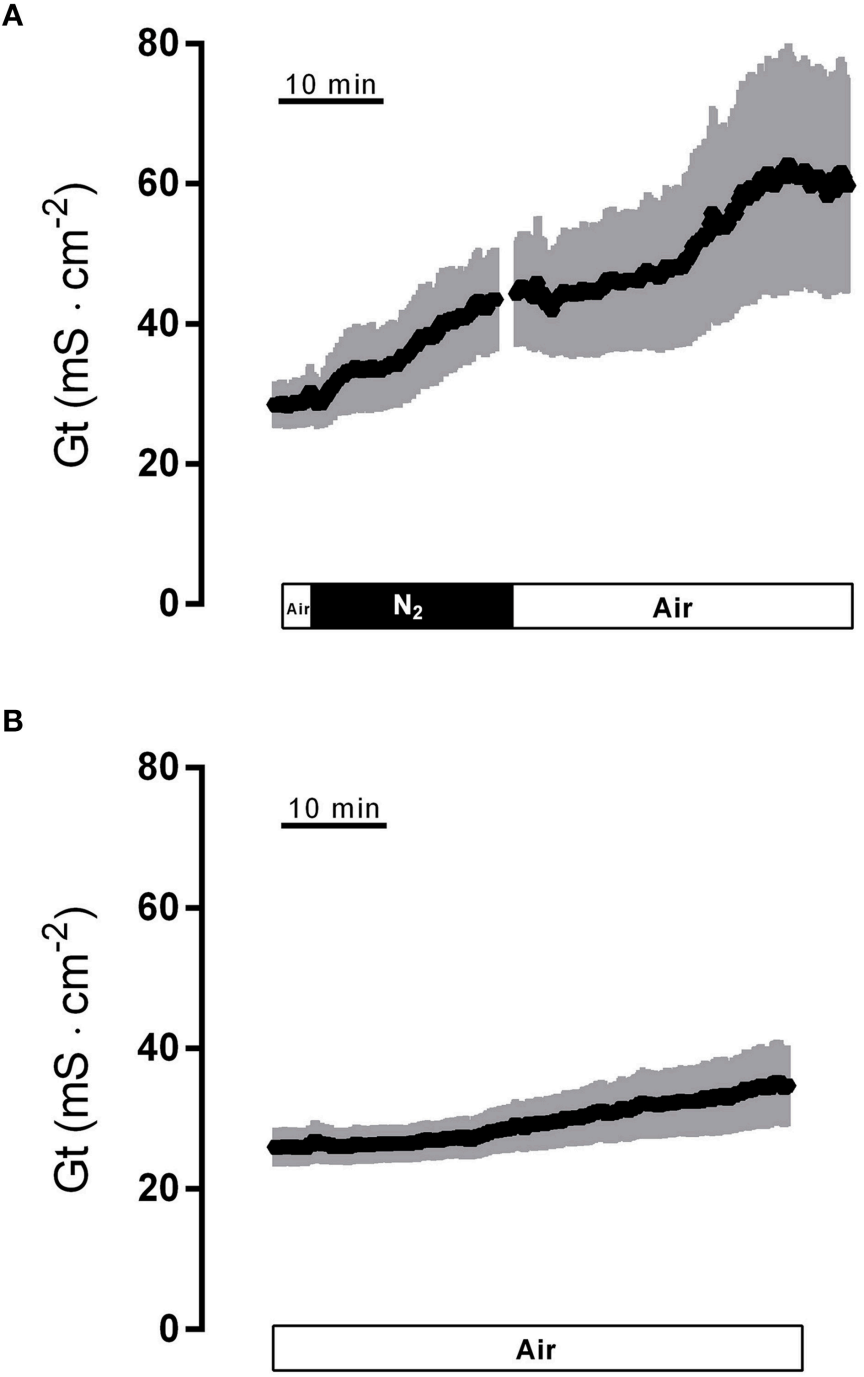
**Effect of hypoxia (N_**2**_; black bar) and reoxygenation on tissue conductance (A) in comparison to a time-dependent control (B) continuously gassed with air (white bar)**. Values are means (symbols) ± SEM (gray area), *n* = 8 **(A)** or 15 **(B)**.

Hypoxia caused a strong increase in tissue conductance (G_t_). It rose from 28.6 ± 3.4 mS·cm^−2^ at the end of the normoxic period to 42.5 ± 7.2 mS·cm^−2^ (*n* = 8, *P* < 0.05) during the hypoxic phase. Switching back to air gassing during the reoxygenation phase did not lead to a recovery of the G_t_, which remained stable at an elevated level for about 15 min, before a secondary rise in G_t_ was observed (Figure 2A). In time-dependent control experiments with continuous air gassing, only a slight increase in G_t_ was observed (Figure 2B) from 26.5 ± 2.8 mS·cm^−2^ to 34.0 ± 5.6 mS·cm^−2^ in the same time interval as described above for the hypoxia/reoxygenation experiments. Due to the strong secondary increase in G_t_ during the late reoxygenation phase, which suggests a damage of the colonic epithelium, in all subsequent experiments the reoxygenation period was limited to 15 min.

### Involvement of K^+^ channels

**Figure 3:**
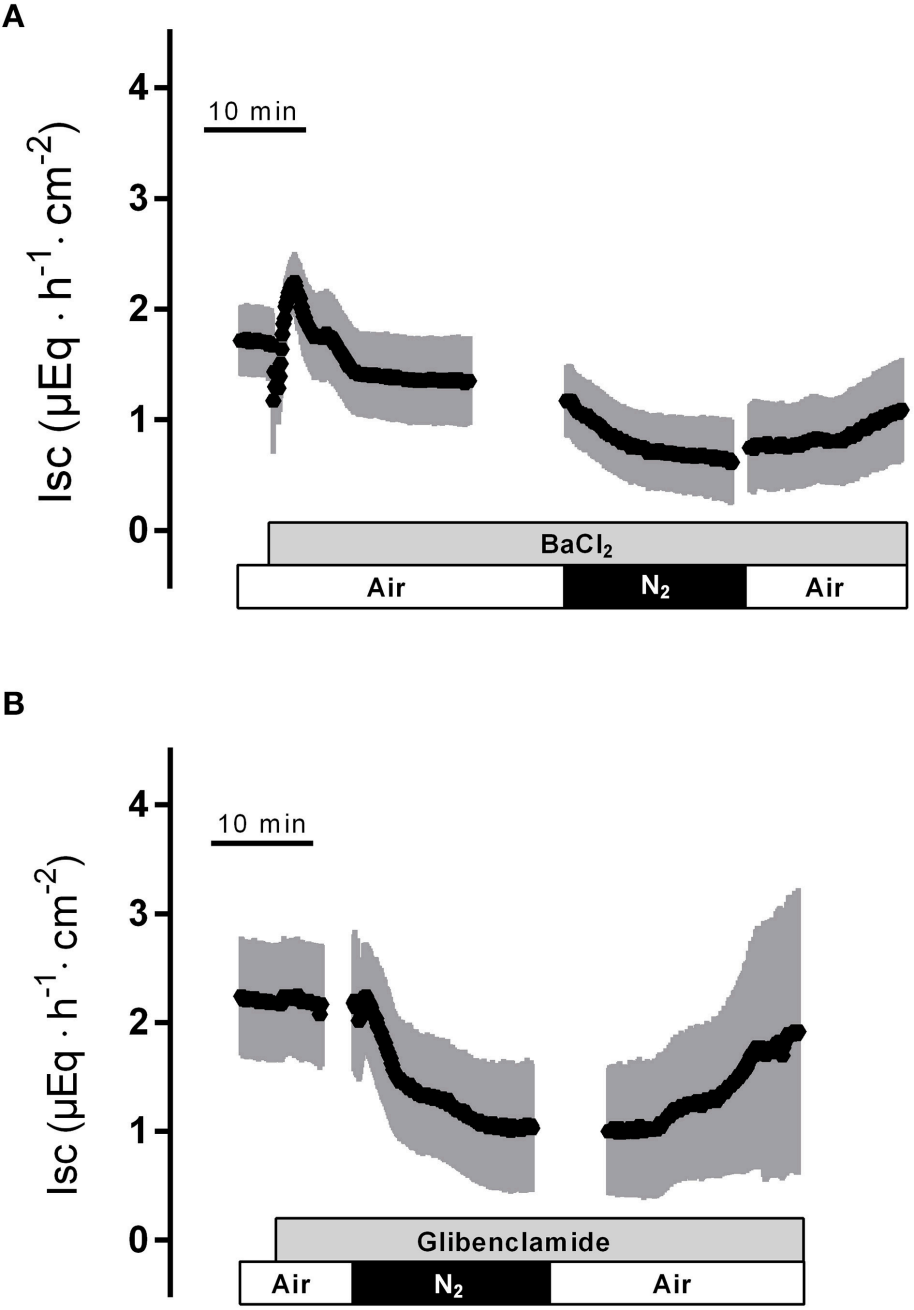
**The nonselective K^**+**^ channel blocker Ba^**2+**^ (10^**−2**^ mol·l^**−1**^ at the serosal side; gray bar) suppresses the peak in I_**sc**_ induced by hypoxia (A), whereas the K_ATP_ channel blocker glibenclamide (5·10^**−4**^ mol·l^**−1**^ at the serosal side) was ineffective (B)**. Values are means (symbols) ± SEM (gray area), *n* = 6–10. For statistics, see Table 1.

To find out whether K^+^ channels play a role in the response to hypoxia and subsequent reoxygenation, blockers of K^+^ conductances known to be involved in rat colonic epithelial ion transport (Strabel and Diener, 1995; Schultheiss and Diener, 1997; Warth and Barhanin, 2003) were used. Preincubation of the tissue with Ba^2+^ (10^−2^ mol·l^−1^ at the serosal side), a nonselective K^+^ channel blocker (Cook and Quast, 1990), caused a significant reduction in the peak of I_sc_ observed during hypoxia (Figure 3A, Table 1). Administration of serosal BaCl_2_ caused a paradox transient increase in I_sc_, which is known to represent the transient activation of Ca^2+^-calmodulin dependent Cl^−^ secretion (Hardcastle et al., 1985).

In order to investigate the involvement of K_ATP_ channels, the tissue was pretreated with glibenclamide (5·10^−4^ mol·l^−1^, at the serosal side), an inhibitor of this type of ${\text{K}}_{}^{+}$channels (for review of the drugs acting on K_ATP_ channels, see Seino and Miki, 2003). However, neither the triphasic change in I_sc_ during hypoxia nor the secondary rise in I_sc_ during the reoxygenation were altered in the presence of glibenclamide (Figure 3A, Table 1). Also preincubation with pinacidil (5·10^−4^ mol·l^−1^, at the serosal side), an opener of K_ATP_ channels, did not alter the currents induced by hypoxia/reoxygenation (Table 1) suggesting that K_ATP_ channels are not involved in the induction of the currents by hypoxia/reoxygenation in this tissue.

### The currents induced by hypoxia/reoxygenation represent changes in Cl^−^ secretion

Changes in I_sc_ across colonic epithelium often represent changes in anion secretion. In order to be secreted, Cl^−^ is taken up across the basolateral membrane via the Na^+^-K^+^-2Cl^−^ cotransporter (NKCC1) and leaves the cell via apical anion channels mainly of the CFTR (cystic fibrosis transmembrane conductance regulator) type (for review see Greger, 2000). The driving for Cl^−^ exit across these channels is the negative membrane potential, which is dominated by a K^+^ diffusion potential generated by K^+^ efflux via basolateral K^+^ channels (Strabel and Diener, 1995; Warth and Barhanin, 2003). In order to find out, whether the changes in I_sc_ observed during hypoxia/reoxygenation represent changes in Cl^−^ transport, blockers of key ion transporters involved in Cl^−^ secretion were used.

**Figure 4:**
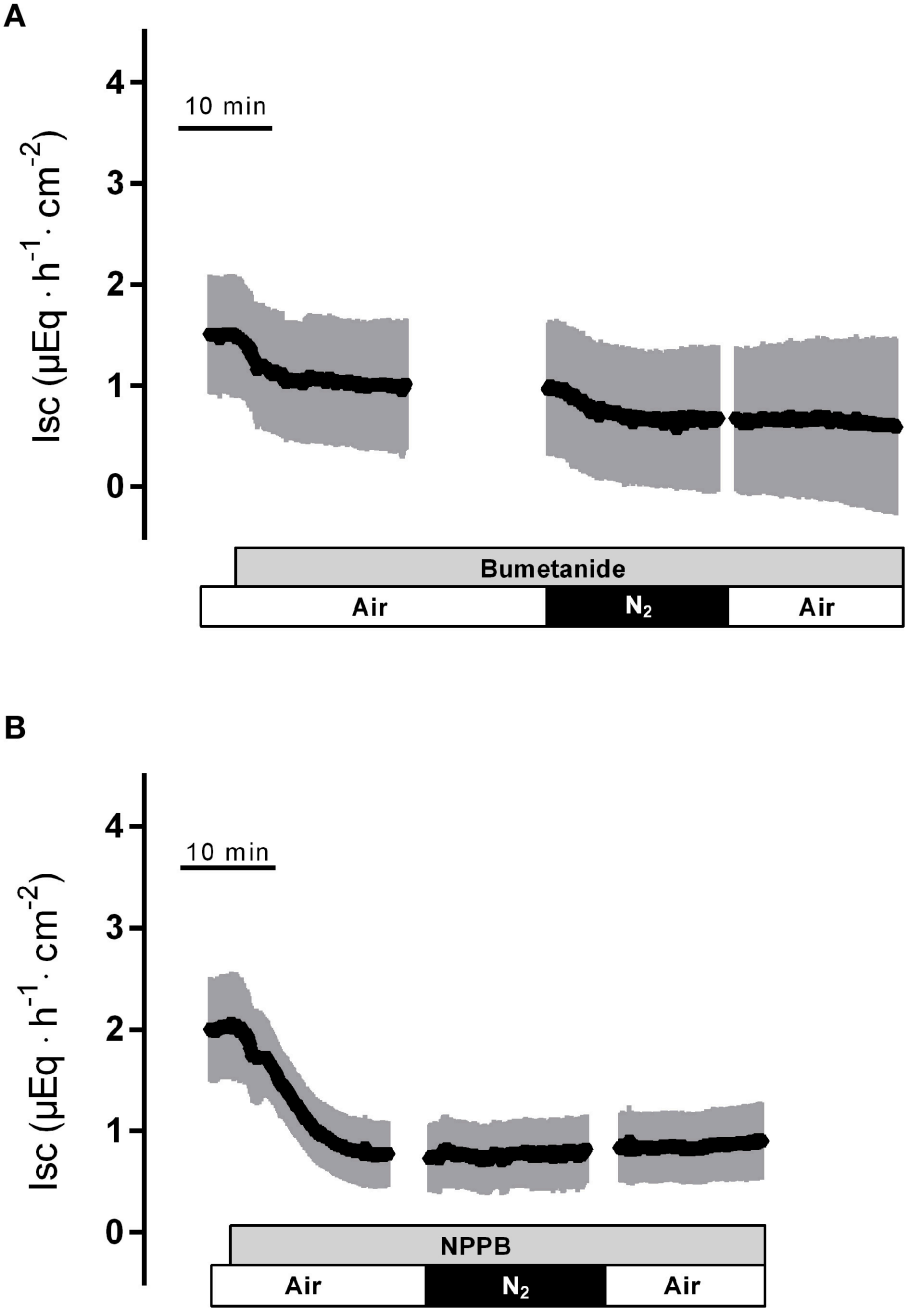
**The response to hypoxia/reoxygenation is suppressed by bumetanide (10^**−3**^ mol·l^**−1**^ at the serosal side; gray bar in A) or NPPB (10^**−4**^ mol·l^**−1**^ at the mucosal side; gray bar in B)**. Values are means (symbols) ± SEM (gray area), *n* = 8–9. For statistics, see Table 2.

In the presence of bumetanide (10^−3^ mol·l^−1^ at the serosal side), an inhibitor of the NKCC1 (for review see Greger, 2000), all three phases of the current response observed during hypoxia were significantly reduced (Table 2). Also the subsequent rise in I_sc_ during the reoxygenation phase was diminished (Figure 4A), although this inhibition did not reach statistical significance (Table 2). In order to block apical Cl^−^ channels, NPPB (10^−4^ mol·l^−1^ at the mucosal side), a Cl^−^ channel blocker (Diener and Rummel, 1989), was used. NPPB suppressed the currents induced by hypoxia and reduced (without reaching statistical significance) the secondary rise in I_sc_ during the reoxygenation period (Figure 4B, Table 2). Both bumetanide as well as NPPB induced a fall in I_sc_, which has to be expected after blockade of basolateral Na^+^-K^+^-2C1^−^ cotransporter and of apical anion channels, respectively, because basal I_sc_ in rat colon is dominated by a spontaneous anion secretion, mainly of Cl^−^ (Strabel and Diener, 1995).

**Table 2:** **The transepithelial currents induced by hypoxia/reoxygenation represent changes in Cl^**−**^ secretion and are blunted by a radical scavenger**.

**Changes in I_sc_ (ΔI_sc_) induced by hypoxia and reoxygenation (**μ**Eq·h^−1^·cm^−2^)**					
**Condition**	**Hypoxia**			**Reoxygenation**	
	**Initial decrease (Dec1) 0–5 min**	**Peak hypoxia 0–5 min**	**Secondary decrease (Dec2) 5–15 min**	**Peak reoxygenation 3–15 min**	***n***
With Cl^−^	−0.91 ± 0.20	0.57 ± 0.17	−1.26 ± 0.19	0.37 ± 0.91	8
Cl^−^-free	−0.39 ± 0.07^*^	−0.08 ± 0.06^*^	−0.47 ± 0.01^*^	0.17 ± 0.07	8
NPPB	−0.14 ± 0.06^*^	0.09 ± 0.05^*^	−0.14 ± 0.09^*^	0.17 ± 0.07	9
Bumetanide	−0.31 ± 0.05^*^	0.04 ± 0.02^*^	−0.49 ± 0.06^*^	0.12 ± 0.12	8
Trolox C	−0.40 ± 0.12	0.15 ± 0.10	−0.55 ± 0.14^*^	0.36 ± 0.06	6

*The effects of hypoxia and reoxygenation on I_sc_ were tested in the absence (Cl^−^ being replaced by the impermeable anion gluconate) of chloride ions, after blockade of Cl^−^ channels with NPPB, after blockade of the Na^+^-K^+^-2C1^−^ cotransporter with bumetanide, or scavenging of oxidants with trolox C. For definitions of the parameters measured, see legend of Table 1. For better orientation, in the 1st row (with Cl^−^) the control response to hypoxia/reoxygenation from Table 1 is shown again. Concentrations of the inhibitors were: NPPB (10^−4^ mol·l^−1^ at the mucosal side), bumetanide (10^−3^ mol·l^−1^ at the serosal side), trolox C (2·10^−4^ mol·l^−1^ at the serosal side). Data are given as change of I_sc_ (ΔI_sc_) compared to the baseline just prior the start of the respective gassing period and are means ± SEM. n = number of tissues*.

* *P < 0.05 vs. control (analysis of variances followed by post-hoc test of Tukey)*.

Substitution of Cl^−^ with the impermeable anion gluconate showed similar effects (Table 2) indicating that hypoxia and subsequent reoxygenation modulate Cl^−^ transport across the colonic epithelium.

### Intracellular messenger substances involved in the changes in I_sc_

The reoxygenation phase following hypoxia mimics the reperfusion situation following ischaemia *in vivo*, which is known to be concomitant with an increased production of oxidants and radicals (Kowaltowski et al., 2009). In order to find out, whether radicals, which are known to act as intracellular signaling molecules (Dröge, 2002), play a role in the currents induced by hypoxia/reoxygenation, tissues were pretreated with trolox C, a derivate of α-tocopherol (Lee et al., 2014; Vergauwen et al., 2015). In the presence of this radical scavenger, the initial decrease and the peak in I_sc_ during the hypoxic phase tended to be reduced and the secondary decrease in I_sc_ was significantly inhibited by 55%. Surprisingly, the increase in I_sc_ during the reoxygenation period remained unaltered in the presence of trolox C (Table 2).

A second messenger known to be involved, e.g., in the response of endothelial cells to hypoxia/reoxygenation is Ca^2+^ (Schäfer et al., 2003). As this second messenger is also able to induce intestinal Cl^−^ secretion predominantly via stimulation of basolateral Ca^2+^-dependent K^+^ channels (Böhme et al., 1991) supported by the transient opening of Ca^2+^-dependent apical Cl^−^ channels (Hennig et al., 2008), the involvement of Ca^2+^ in the I_sc_ response evoked by hypoxia/reoxygenation was investigated.

In the absence of serosal Ca^2+^, the peak in I_sc_ during the early hypoxic period was significantly reduced suggesting that this phase is caused by the transient stimulation of Ca^2+^-dependent Cl^−^ secretion. When in addition the release of Ca^2+^ from intracellular stores was inhibited with 2-APB (10^−4^ mol·l^−1^), a blocker of inositol-1,4,5-trisphosphate (IP_3_) receptors (Maruyama et al., 1997), all three phases of the I_sc_ response during hypoxia were significantly diminished (Table 3).

**Table 3:** **Role of Ca^**2+**^ in the hypoxia/reoxygenation-evoked changes in I_**sc**_**.

**Changes in I_sc_ (ΔI_sc_) induced by hypoxia and reoxygenation (μEq·h^−1^·cm^−2^)**					
**Condition**	**Hypoxia**			**Reoxygenation**	
	**Initial decrease (Dec1) 0–5 min**	**Peak hypoxia 0–5 min**	**Secondary decrease (Dec2) 5–15 min**	**Peak reoxygenation 3–15 min**	***n***
With Ca^2+^	−0.91 ± 0.20	0.57 ± 0.17	−1.26 ± 0.19	0.37 ± 0.91	8
0 Ca^2+^	−0.86 ± 0.14	0.20 ± 0.05^*^	−0.94 ± 0.15	0.50 ± 0.07	13
0 Ca^2+^+ 2-APB	−0.20 ± 0.06^*^	0.05 ± 0.02^*^	−0.28 ± 0.10^*^	0.21 ± 0.05	8

*The effects of hypoxia and reoxygenation on I_sc_ were tested in the presence (1st row) or absence of Ca^2+^ either alone (2nd row) or combined with 2-APB (3rd row; 10^−4^ mol·l^−1^ at the serosal side). For definitions of the parameters measured, see legend of Table 1. For better orientation, in the 1st row (with Ca^2+^) the control response to hypoxia/reoxygenation (Table 1) is shown again. Ca^2+^ was omitted from the serosal side of the tissue. Data are given as change of I_sc_ (ΔI_sc_) compared to the baseline just prior the start of the respective gassing period and are means ± SEM. n = number of tissues*.

* *P < 0.05 vs. control (analysis of variances followed by post-hoc test of Tukey)*.

### Changes in the cytosolic Ca^2+^ concentration

**Figure 5:**
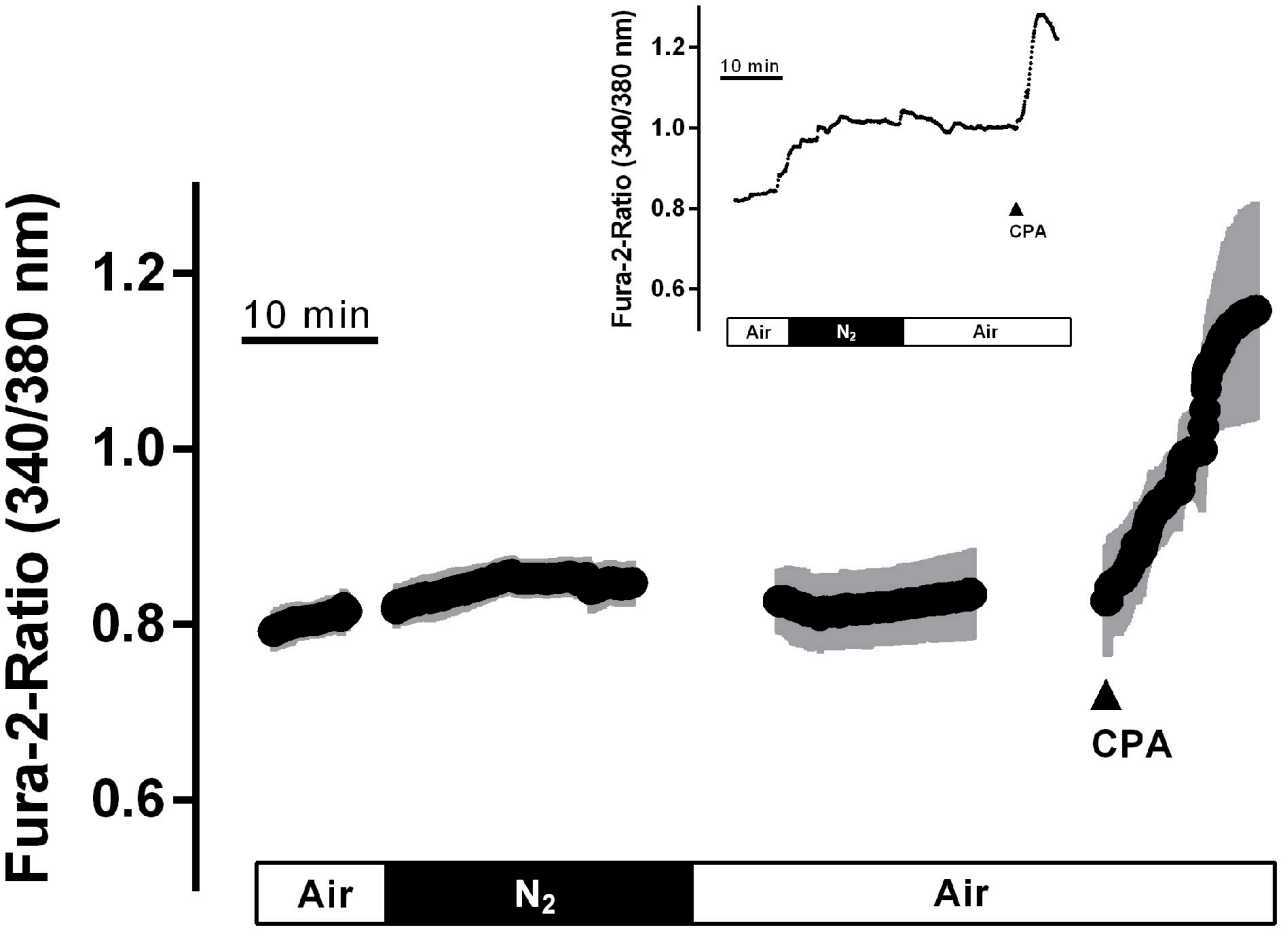
**In fura-2 imaging experiments with isolated crypts a moderate increase in the cytosolic Ca^**2+**^ concentration during hypoxia was observed followed by a secondary increase during the reoxygenation phase**. At the end of the experiment, cyclopiazonic acid (CPA; 10^−5^ mol·l^−1^; filled arrow) was administered as viability control. Values are means (symbols) ± SEM (gray area), *n* = 40. The inset shows an original tracing which exemplifies the increase in the fura-2 ratio signal more clearly, as the time ensemble presented in the averaged figure tends to dampen signal amplitudes if not all cells respond synchronously. For statistics, see Table 4.

Ca^2+^ measurements at fura-2-loaded isolated crypts in imaging experiments revealed a slow increase of the cytosolic Ca^2+^ concentration during hypoxia (Figure 5). When switching back to the perfusion of the chamber with air-gassed buffer solutions, the fura-2 ratio signal further increased (Table 4). At the end of the experiment, intracellular Ca^2+^ stores were depleted by cyclopiazonic acid (10^−5^ mol·l^−1^), an inhibitor of sarcoplasmic-endoplasmic Ca^2+^-ATPases (SERCA; Plenge-Tellechea et al., 1997), which served as viability control. Cyclopiazonic acid induced a consistent and prompt increase in the fura-2 ratio signal thus excluding irreversible changes of the colonic epithelium during the hypoxic period. The increase in the cytosolic Ca^2+^ concentration was abolished when the crypts were superfused with a Ca^2+^-free solution either alone or in combination with the IP_3_ receptor blocker 2-APB (10^−4^ mol·l^−1^). The same was observed, when the crypts were pretreated with Gd^3+^ (5·10^−6^ mol·l^−1^), a blocker of nonselective cation channels (Frings et al., 1999) responsible for store-operated Ca^2+^ entry in these cells (Table 4).

**Table 4:** **Changes in the cytosolic Ca^**2+**^ concentration of isolated crypts during hypoxia/reoxygenation**.

	**Δ fura-2 ratio (340/380 nm)**			
**Condition**	**Hypoxia (means measured over the final 3 min)**	**Reoxygenation (means measured over the final 3 min)**	**Cyclopiazonic acid (peak response)**	***n***
Control	0.0008 ± 0.01	0.03 ± 0.02^*^	0.22 ± 0.03	40
Trolox C	−0.07 ± 0.32^*^	0.00 ± 0.42	0.42 ± 0.57	46
Gd^3+^	−0.01 ± 0.01	−0.01 ± 0.02	0.3 ± 0.02^*^	57
0 Ca^2+^	−0.10 ± 0.01^*^	−0.11 ± 0.03^*^	0.17 ± 0.01	60
0 Ca^2+^+ 2-APB	0.02 ± 0.01	−0.001 ± 0.01	0.08 ± 0.005	36

Changes in the fura-2 ratio signal induced by hypoxia and subsequent reoxygenation under control conditions (with Ca^2+^), in the presence of the radical scavenger trolox C (10^−4^ mol·l^−1^), after blockade of store-operated Ca^2+^ channels with Gd^3+^ (5·10^−6^ mol·l^−1^), in the absence of Ca^2+^ ions or after additional blockade of IP_3_ receptors with 2-APB (10^−4^ mol·l^−1^). Data are given as change of fura-2 ratio [Δfura-2 ratio (340/380 nm)] compared to the baseline just prior the start of the respective gassing period or prior administration of cyclopiazonic acd 10^−5^ mol·l^−1^) and are means ± SEM. n = number of tissues

* *P < 0.05 vs. control (analysis of variances followed by post-hoc test of Tukey)*.

When the crypts were pretreated with the radical scavenger trolox C (10^−4^ mol·l^−1^), no increase in the fura-2 ratio was induced by hypoxia and also the secondary increase during the reoxygenation period was abolished (Table 4).

### Involvement of reactive oxygen species

**Figure 6:**
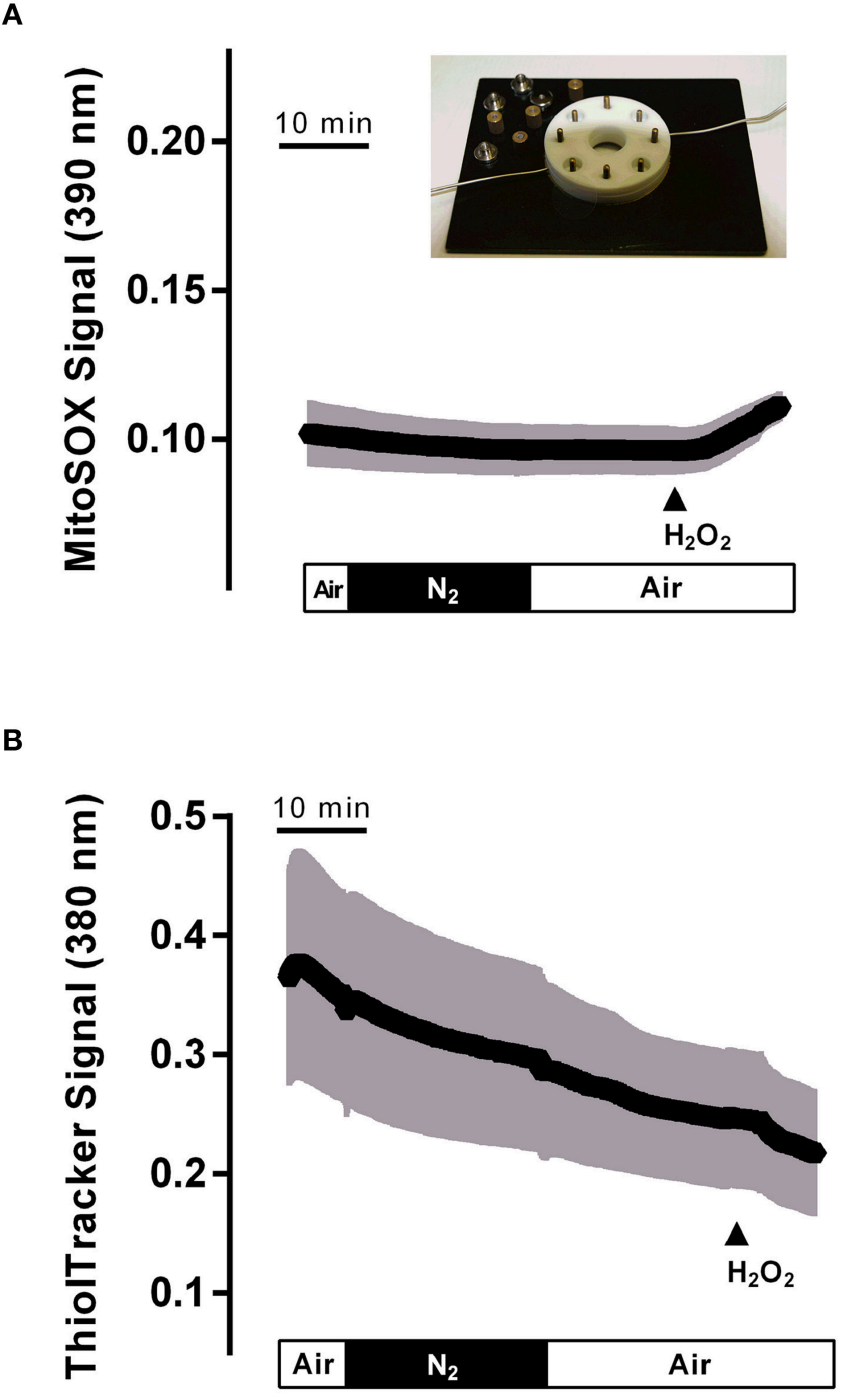
**Hypoxia (N_**2**_ gassing; black bar)/reoxygenation did not affect mitochondrial radical production (A) or cytosolic glutathione concentration (B) in isolated colonic crypts**. Values are means (symbols) ± SEM (gray area), *n* = 75–90. At the end of the experiments, H_2_O_2_ (10^−2^ mol·l^−1^; filled arrow) was administered as control. The inset shows the hypoxia chamber, in which the crypts (mounted between two glass slides fixed between teflon rings) were superfused with N_2_- or air-gassed solutions.

As the production of oxidants in mitochondria starts with the conversion of the superoxide anion (${\text{O}}_{2}^{-}$•) to hydrogen peroxide (H_2_O_2_), a reaction catalyzed by the enzyme superoxide dismutase (Yu, 1994), it was of interest to check the production of potential oxidants in the isolated crypts during hypoxia/reoxygenation. For this purpose, the fluorescent dye mitosox was used. However, the mitosox signal was unaltered during the hypoxia and the subsequent reoxygenation period (Figure 6A). The mitosox fluorescence (arbitrary units) fell by −0.005 ± 0.002 during the hypoxia and by −0.004 ± 0.002 (*n* = 50) during reoxygenation. In time-dependent control experiments (without hypoxia/reoxygenation), a similar change of the mitosox signal by −0.005 ± 0.002 and by −0.004 ± 0.003 (*n* = 44) in the same time intervals was observed. At the end of the experiment H_2_O_2_ (10^−2^ mol·l^−1^) was administered in order to prove the validity of the method. As expected, hydrogen peroxide induced a strong increase in the mitosox fluorescence signal by 0.12 ± 0.004 (*n* = 50) in the hypoxia group and by 0.16 ± 0.01 (*n* = 44) in the time-dependent control experiments.

Physiological protection against oxidants is insured by glutathione (GSH), the main reducing agent in the cytosol (Mandavilli and Janes, 2010), which keeps thiols in the reduced state in intact cells. In order to find out, whether cytosolic glutathione might “trap” the expected production of oxidants during hypoxia/reoxygenation, changes in the cytosolic glutathione concentration were monitored by loading the colonic crypts with thiol tracker. However, no change in the signal of this fluorescent indicator was observed during the hypoxic period, i.e., during superfusion with N_2_-gassed solutions, or the subsequent reoxygenation period. Instead, a continuous, time-dependent decrease of the signal, which probably reflects bleaching of the dye, was observed (Figure 6B). The thiol tracker signal (arbitrary units) fell from the beginning to the end of the hypoxia period by −0.06 ± 0.009 and by −0.11 ± 0.01 (*n* = 90) during the subsequent reoxygenation phase. These values were not different from the changes in time-dependent control experiments, where the thiol tracker fluorescence fell by −0.07 ± 0.005 and −0.09 ± 0.006 (*n* = 75) in the respective time periods. At the end of each experiment, H_2_O_2_ (10^−2^ mol·l^−1^) was administered. Hydrogen peroxide induced a prompt decrease of the thiol tracker signal by −0.04 ± 0.005 (*n* = 90) in the hypoxia experiments and by −0.04 ± 0.005 (*n* = 75) in the time-dependent control experiments, as has to be expected after challenging the cell with oxidants.

## Discussion

The intestinal epithelium is strongly affected by changes in oxygen supply, which has an influence on the expression, cellular localization or activity of several transporters for ions and nutrients in the gut (Ward et al., 2014). In the present study, we observed a triphasic change in electrogenic ion transport measured as I_sc_ across rat distal colon during hypoxia: a transient decrease followed by a short rise in I_sc_, before the current finally fell below the initial baseline (Figure 1A). Anion substitution experiments (Table 2) and experiments in which Cl^−^ secretion was blocked either by inhibition of the Na^+^-K^+^-2Cl^−^-cotransporter (Figure 4A), which represents the dominant Cl^−^ uptake mechanism across the basolateral membrane (Russell, 2000), or inhibition of Cl^−^ channels (Figure 4B) responsible for Cl^−^ exit across the apical membrane (Greger, 2000), revealed that these changes in I_sc_ represent changes in Cl^−^ secretion.

The transient increase in I_sc_ (“peak” in Figure 1A) thus represents a short-lasting stimulation of anion secretion. Chloride secretion is under the control of intracellular second messengers such as Ca^2+^, cAMP and cGMP (Binder and Sandle, 1994). The secretory response was inhibited in the absence of serosal Ca^2+^, especially when this maneuver was combined by the additional presence of 2-ABP blocking the release of intracellularly stored Ca^2+^ via IP_3_ receptors (Table 4). Furthermore, hypoxia leads to modest, but consistent increase in the cytosolic Ca^2+^ concentration of isolated colonic crypts loaded with the Ca^2+^-sensitive dye fura-2 (Figure 5). Therefore, this current probably reflects a stimulation of Ca^2+^-dependent Cl^−^ secretion during hypoxia. The dominant mechanism, by which an increase in the cytosolic Ca^2+^ concentration stimulates Cl^−^ secretion is the activation of Ca^2+^-dependent basolateral K^+^ channels, which hyperpolarizes the membrane and thereby increases the driving force for Cl^−^ exit across apical anion channels (Böhme et al., 1991; Strabel and Diener, 1995). This process is supported by the transient opening of Ca^2+^-dependent Cl^−^ channels in the apical membrane (Hennig et al., 2008). Indeed, in human colonic epithelium chemically induced hypoxia has been shown to activate intermediate conductance Ca^2+^-dependent K^+^ channels (Loganathan et al., 2011). An increase in the cytosolic Ca^2+^ concentration will also stimulate apical Ca^2+^-dependent K^+^ channels, which are found in the brushborder membrane, too (Schultheiss and Diener, 1997). This may underlie the Ca^2+^-dependent (Table 3) initial fall in I_sc_ (“Dec1” in Figure 1A) during hypoxia preceding the transient Cl^−^ secretion leading to a rise in I_sc_. How these changes in ion transport are initiated is finally unclear. However, during hypoxia epithelial cells release adenosine as shown on T84 cells (Matthews et al., 1995), a human colonic tumor cell line. Thus, paracrine mediators may be involved in the control of epithelial ion channel activity during hypoxia/reoxygenation.

The central role of K^+^ channels in the initial response to hypoxia is underlined by the action of Ba^2+^, which suppressed the peak in I_sc_ during hypoxia (Figure 3A) and tended to reduce the initial fall in I_sc_ at the onset of hypoxia (Table 2). Surprisingly, despite functional, morphological and molecular biological evidence demonstrating the expression of ATP-sensitive K^+^ channels in the basolateral membrane of rat colonic epithelium (Pouokam et al., 2013), neither blockade of these channels with glibenclamide (Figure 3B) nor activation with pinacidil prior the onset of hypoxia had any effect on the electrogenic response evoked by hypoxia or reoxygenation. So despite the expected fall in the cytosolic ATP concentration after impairment of mitochondrial oxidative phosphorylation during hypoxia, K_ATP_ channels do not contribute to the changes in colonic ion transport during hypoxia. This clearly contrasts the response of the epithelium from that of excitable tissues, where these channels represent an important protective mechanism via hyperpolarization of the membrane, which finally reduces excitability and thereby the energy demand of these cells (Hibino et al., 2010). Consequently, in colonic epithelia K_ATP_ channels must exert other functions, such as to sense the gasotransmitter H_2_S (Pouokam and Diener, 2011).

The final response, however, of the epithelium to hypoxia consists in a long-lasting fall in I_sc_ (Figure 1A) consistent with an inhibition of anion secretion. Several mechanisms may underlie this reduction in transepithelial current. For example, in T84 cells a decrease in the intracellular level of cGMP and cAMP, i.e., 2 s messengers responsible for activation of transepithelial Cl^−^ secretion, has been observed during hypoxia (Taylor et al., 1998b). Also the activation of an AMP kinase during hypoxia is known to downregulate especially cAMP-induced anion secretion (Collins et al., 2011). Furthermore, already a reduction in the cytosolic ATP concentration alone might directly impair the opening of the CFTR channel, the dominant Cl^−^ channel in the apical membrane (Greger, 2000), as this member of the ABC (ATP-binding cassette) protein family needs phosphorylation for activation and in addition ATP hydrolysis for its gating (Gadsby et al., 2006). On the long-term, there seems even to be a downregulation of this channel on the transcriptional level during hypoxia via the HIF-1 (hypoxia-inducible factor-1) pathway as shown in the colonic cells lines T84 and Caco-2 (Zheng et al., 2009).

Concomitant with the change in I_sc_, there is a strong increase in tissue conductance, i.e., in the ionic permeability, of the epithelium during hypoxia, which even further rises after a short delay during the reoxygenation period (Figure 2A). A small increase in G_*t*_ was also observed in the time-dependent control experiments (Figure 2B). This is probably due to the experimental design, in which we decided to switch between “normoxic” conditions (i.e., gassing with room air containing 20.9% (v/v) O_2_ instead of pure oxygen), and N_2_ gassing, to mimic better the *in vivo* situation of ischemia/reperfusion. Obviously, this seems to limit the normally observed “longevity” of colonic specimens in Ussing chamber setups, which are usually gassed with 95–100% (v/v) O_2_. In T84 cells, a release of epithelial cytokines such as tumor necrosis factor-α has been shown to play a role in the increase in G_t_ during hypoxia, which probably reflects an increased permeability of the tight junctions (Taylor et al., 1998a). However, this does not necessarily represent only an unspecific damage of the epithelium due to hypoxia, but may in addition involve a regulated process as inhibition of Ca^2+^-dependent K^+^ channels has been shown to reduce the increase in the paracellular permeability during energy depletion in human colon (Loganathan et al., 2011).

Experiments with the radical scavenger trolox C suggest that both the long-lasting inhibition of I_sc_ during the late hypoxia period (Table 2) as well as the rise of the cytosolic Ca^2+^ concentration during this period (Table 4) involved reactive oxygen species. However, no changes could be detected in the fluorescence of the superoxide anion-sensitive dye mitosox (Figure 6A) or the thiol-sensitive dye thiol tracker (Figure 6B) indicating that the expected increase in ${\text{O}}_{2}^{-}$• or a compensatory fall in the cytosolic glutathione level were below the level of detection and at least much smaller compared to the changes induced by a strong oxidant such as H_2_O_2_. *In vivo*, the situation is most likely different due to the infiltration of the intestinal wall with neutrophil granulocytes, which are a strong source of oxidants (Gonzalez et al., 2015). On the other hand, morphological studies performed at rat intestine suggest that ischemia causes only a modest histological damage in the colon in comparison to small intestine (Leung et al., 1992). Especially, in contrast to the small intestine, colonic mucosal damage was not enhanced by reperfusion, which might correlate with the low level of xanthine oxidase activity (about only 10% of that observed in small intestine) and thus a smaller production rate of reactive oxygen species in this segment of the gut (Leung et al., 1992). This suggests a relative high capacity of the colonic epithelium with its low O_2_ partial pressure even under physiological conditions (Zheng et al., 2015) to deal with enhanced radical production during hypoxia/reoxygenation.

The colonic epithelium is exposed to daily fluctuations of nutrient contents, microflora and mesenteric blood circulation. Adaptive mechanisms have been developed for facing such threats. Chloride secretion may be a “priming” consequence of epithelial cells which are regularly exposed to stimuli like inflammation correlated with hypoxia, which often is associated clinically with diarrhea (Zheng et al., 2009; Ward et al., 2014). The goal of this secretion could be “flushing” pathogens from the epithelium as if a microbial assault was running.

## Author contributions

Conception and design of the work (SS, EP, MD); acquisition (SS, EP), analysis and interpretation of data (SS, EP, MD); drafting, revising and final approval the manuscript (SS, EP, MD).

### Conflict of interest statement

The authors declare that the research was conducted in the absence of any commercial or financial relationships that could be construed as a potential conflict of interest.
